# Clinical Remarks

**Published:** 1867-08

**Authors:** 


### ARTICLE V.

### *Clinical Remarks*

### On "Schmidt's" disease of the Lower Jaw. At Surgeon Nelaton's Clinique. Paris.

*Case.*—Man aged 40 years. German—has been a worker in a match factory—his part of the work being to mix the paste formed of phosphorus and chlorate of potash, in which to dip the stems. This affection is especially common in Germany and its provinces, where the Congreve and lucifer matches are made on a large scale. The disease is produced by inhaling the vapors of phosphorus. The employees of the match factories are chiefly liable to it. It is really a necrosis of the inferior maxillary bone, After several months working over the warm phosphorus, the patient first feels a constant pain in the lower jaw, and ascribes it to some disease of one of the molar teeth of that side; though there may be and most often is no decay in it, the tooth is removed without relief to the pain in the jaw. This pain may be irregular or periodical—but it yields to no ordinary treatment. Soon necrosis of a portion of the bone becomes apparent, and a sequestrum is discovered, either in part or wholly detached—indeed it may fall out without any surgical interference whatever. When the latter occurs fistulous openings result, and are often long in closing. Strange to state, this disease is confined to the lower jaw, and is known only in workers in the paste referred to, and not in the hands engaged in other work in the same factory—nor in those whose business it is to manufacture phosphorus from bones for purposes of commerce. The obvious treatment is an early change of occupation and removal of the diseased sequestrum, with a course of tonics, good soft diet, and plenty of out-door exercise. A. A.

---

